# The bronchial epithelial cell bacterial microbiome and host response in patients infected with human immunodeficiency virus

**DOI:** 10.1186/s12890-016-0303-4

**Published:** 2016-11-09

**Authors:** Marc A. Sze, Stella Xu, Janice M. Leung, Emily A. Vucic, Tawimas Shaipanich, Aida Moghadam, Marianne Harris, Silvia Guillemi, Sunita Sinha, Corey Nislow, Darra Murphy, Cameron Hague, Jonathon Leipsic, Stephen Lam, Wan Lam, Julio S. Montaner, Don D. Sin, S. F. Paul Man

**Affiliations:** 1 Centre for Heart Lung Innovation, St. Paul’s Hospital & Department of Medicine, University of British Columbia, Rm 166 – 1081 Burrard St., Vancouver, BC V6Z 1Y6 Canada; 2 Department of Integrative Oncology, BC Cancer Research Centre, Vancouver, BC Canada; 3 Division of Respiratory Medicine, St. Paul’s Hospital, University of British Columbia, Vancouver, BC Canada; 4 AIDS Research Program, St. Paul’s Hospital, Vancouver, BC Canada; 5 Department of Family Medicine, Faculty of Medicine, University of British Columbia, Vancouver, BC Canada; 6 Division of HIV/AIDS, Department of Medicine, University of British Columbia, Vancouver, BC Canada; 7 Faculty of Pharmaceutical Sciences, Pharmaceutical Sciences Building, University of British Columbia, Vancouver, BC Canada; 8 Department of Radiology and Diagnostic Imaging, St. Paul’s Hospital, Vancouver, BC Canada; 9 British Columbia Centre for Excellence in HIV/AIDS, St. Paul’s Hospital, Vancouver, BC Canada

**Keywords:** Bronchial brushing, Bacterial microbiome, HIV, Lungs, Gene expression

## Abstract

**Background:**

Chronic Obstructive Pulmonary Disease (COPD) is an important comorbidity in patients living with human immunodeficiency virus (HIV). Previous bacterial microbiome studies have shown increased abundance of specific bacterium, like *Tropheryma whipplei*, and no overall community differences. However, the host response to the lung microbiome is unknown in patients infected with HIV.

**Methods:**

Two bronchial brush samples were obtained from 21 HIV-infected patients. One brush was used for bacterial microbiome analysis using the Illumina MiSeq^TM^ platform, while the other was used to evaluate gene expression patterns of the host using the Affymetrix Human Gene ST 2.0 array. Weighted gene co-expression network analysis was used to determine the relationship between the bacterial microbiome and host gene expression response.

**Results:**

The Shannon Diversity was inversely related to only one gene expression module (*p* = 0.02); whereas evenness correlated with five different modules (*p* ≤ 0.05). After FDR correction only the Firmicutes phylum was significantly correlated with any modules (FDR < 0.05). These modules were enriched for cilia, transcription regulation, and immune response. Specific operational taxonomic units (OTUs), such as OTU4 (Pasteurellaceae), were able to distinguish HIV patients with and without COPD and severe emphysema.

**Conclusion:**

These data support the hypothesis that the bacterial microbiome in HIV lungs is associated with specific host immune responses. Whether or not these responses are also seen in non-HIV infected individuals needs to be addressed in future studies.

**Electronic supplementary material:**

The online version of this article (doi:10.1186/s12890-016-0303-4) contains supplementary material, which is available to authorized users.

## Background

The increased susceptibility of patients infected with human immunodeficiency virus (HIV) to lung diseases, in particular chronic obstructive pulmonary disease (COPD), has now been recognized in numerous epidemiological studies [1–3]. Because cigarette smoke exposure only partially explains this elevated risk [1], the pathogenesis of comorbid lung disease in HIV is largely a mystery. Investigation of the HIV lung bacterial microbiome using bronchoalveolar lavage fluid has suggested a greater abundance of *Tropheryma whipplei* in the HIV lung [4] and no significant impact of anti-retroviral therapy on the bacterial community composition in HIV-infected individuals [5]. However, the impact of the lung microbiota on the pathogenesis of chronic lung diseases such as COPD in HIV is unclear. Moreover, there is little information whether the lung microbiome is associated with significant host responses in the lungs.

Although characterization of bacterial community composition in a disease state is an important first step in uncovering the possible clinical relevance of the lung microbiome [4, 6], the next logical step is to discover whether or not changes in the lung microbiome induce a host response that may be important in disease pathogenesis. We have recently shown, using lung tissue samples from non-HIV infected individuals with COPD, that shifts in the lung microbiome are associated with important changes in inflammatory response in these lungs [7]. One important limitation of that study was that the microbiome was characterized in a block of lung tissue and as such cell-specificity could not be ascertained. Moreover, this study did not include any patients with HIV infection. Here, we extend these observations by investigating the interactions between the host gene expression response and the bacterial microbiome in bronchial epithelial cells of small airways collected from the same site in patients infected with HIV. The specific aims of this study were to describe the bacterial community composition of the HIV bronchial epithelium and to determine whether the bacterial microbiome of the HIV bronchial epithelium is associated with specific gene expression signatures of the host that may reveal the underlying pathogenesis of chronic airways disease in HIV-infected individuals.

## Methods

### Patient population

All subjects provided written informed consent for the collection of cytologic brushings for research purposes under the UBC Providence Health Care ethics protocol H14-03267. Subjects were recruited from patients undergoing bronchoscopy for pulmonary nodules, masses, or pneumonia (all conditions were diagnosed radiographically by computed tomography (CT) imaging at St. Paul’s Hospital, Vancouver, BC). Entry criteria into the study included documented HIV-1 infection and ≥19 years of age. All subjects performed spirometry according to the American Thoracic Society/European Respiratory Society guidelines [8] within three months, except for five subjects who underwent bronchoscopy for acute infection. COPD was defined by post-bronchodilator forced expiratory volume in one second (FEV1)/forced vital capacity (FVC) ratio of less than 70 %.

Patients underwent thoracic CT imaging using a 64 detector CT scanner (Discovery HD 750 or a VCT, GE Healthcare, Milwaukee, WI). A central imaging core laboratory (SPH CT Corelab), blinded to spirometry and clinical data, interpreted the CT images for emphysema based on a modified method of Kazerooni, et al. [9]. Emphysema severity was qualitatively scored according to an established algorithm (see Additional file 1). CT scans were also qualitatively scored for respiratory bronchiolitis (none, trivial, mild, moderate, and severe) and bronchiectasis (presence or absence). Details on bronchoscopy and specimen collection can be found in the Additional file 1. Bronchial epithelial cells were obtained from sites away from the acute infection, masses or nodules.

### Bacterial microbiome analysis

DNA was extracted using the Qiagen DNeasy Blood and Tissue Kit (Qiagen, Toronto, Ontario) from both patient samples and background negative environmental controls. Total 16S load was quantified using a droplet digital polymerase chain reaction (ddPCR) assay [10]. These background controls were used to assess whether the bacterial community of the HIV samples were impacted by the instruments and reagents used during the extraction and PCR process. To assess the 16S load within the samples the average 16S load from the negative controls were subtracted from each HIV 16S sample. Touchdown PCR [11] of the 16S rRNA gene V4 region was used to generate a DNA template for sequencing. Cycle conditions for the touchdown PCR can be found in the Additional file 1. Sequencing was performed on an Illumina MiSeq^TM^ (Illumina, Redwood City, CA, USA) with 2 × 250 paired end-read chemistry. The protocol established by Kozich, et al. was used for the sequencing and subsequent data cleanup within the program mothur (V1.34.4) [12]. After processing, sequence cleanup, and chimera removal, a total of 3,559,398 reads remained. Data analysis was performed in R (V3.2.0) and R studio (V0.99.441) employing the vegan (V2.3-0) package [13, 14]. In order to adequately perform alpha and beta diversity analysis subsampling to the lowest total reads (3164) was performed [15]. Along with a 97 % similarity threshold, a total of 451 different Operational Taxonomic Units (OTUs) were identified. Sequence data has been deposited in the NCBI sequence read archive under the accession number SRP068430. The corresponding metadata can be found at http://www.ncbi.nlm.nih.gov/Traces/study/?acc=SRP068430&go=go. Alternatively, one can use the following link http://www.ncbi.nlm.nih.gov/Traces/study/ and search for SRP068430 to bring up the relevant information needed. The relevant samples used for this study are BIDC1-4, BIDC7-10, BIDC14-26 for the HIV samples and Neg1-4 for the background negative control samples.

### Microarray analysis

RNA was extracted using the Qiagen RNeasy Plus Universal Kit (Qiagen, Toronto, Ontario). 1 ug of RNA was processed and hybridized onto the Affymetrix Human Gene ST 2.0 array (Affymetrix Inc, Santa Clara, USA) according to the manufacturer’s protocol at the Hospital for Sick Children, Centre for Applied Genomics (Toronto, Ontario). Raw CEL files were processed and RMA normalized in R (V3.2.0) and R studio (V0.99.441) using a standard protocol from the *oligo* package (V1.32.0) [16]. Gene symbols and names were obtained from the hugene20sttranscriptcluster.db from bioconductor [17].

### Data analysis and integration

For phyla level comparisons a *t*-test with Bonferroni correction was applied. A random forest algorithm with Boruta feature selection [18, 19] was used to identify any OTUs that could be discriminative of specific clinical traits in the patient population (e.g. smoking status, COPD, etc.). Traits were chosen for Boruta feature selection analysis based on whether or not their PERMANOVA value was less than or equal to 0.1. We determined differences in the bacterial community composition between groups by a Bonferroni corrected PERMANOVA [20] of ≤ 0.0125. Robustly co-expressed sets of genes (i.e. modules) were identified in airway expression data using a weighted gene co-expression network analysis (WGCNA) [21, 22]. Modules eigengene vector values were then compared to alpha diversity measures (Shannon Diversity, OTU richness, and evenness) and phyla measures using the WGCNA (V1.46) R package [21, 22]. For this analysis no grouping was performed by smoking, CD4 cell count, or viral load status as these variables were not significantly associated with microbiome measures. The Database for Annotation, Visualization, and Integrated Discovery (DAVID) [23] was used to identify the most relevant pathway clusters for each module that were significantly correlated to the specific bacterial microbiome measurements. The top 10 enrichment clusters were used as a guide to discovering pathways that were most strongly associated with each module. A false discovery rate (FDR) of less than 0.05 was considered significant. In addition to this module comparison versus microbiome metrics for our network used p-values of less than 0.05 as well. The OTU data was reported to the lowest taxonomic identification, either within the text or in the respective figure.

## Results

### Overview of the bacterial microbiome in the HIV cohort

**Fig. 1:**
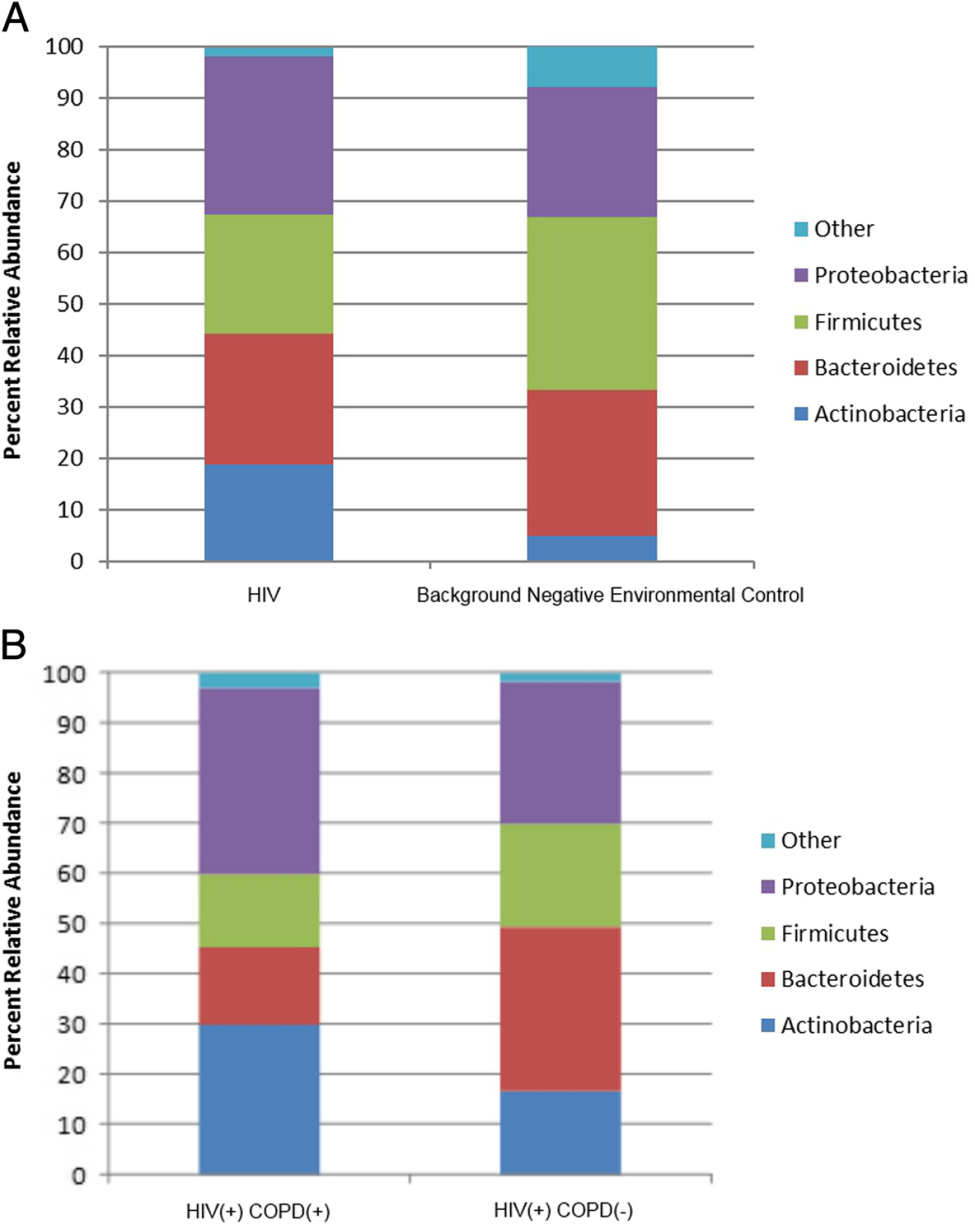
Breakdown of major phyla. **a** Comparison between HIV patient samples (*n* = 21) and background negative controls (*n* = 4). There was a significant difference in the Actinobacteria phylum between HIV and background negative controls (*p* = 0.003). There were also slightly more Proteobacteria in the HIV group than in the background negative controls (*p* > 0.05). **b** Comparison between HIV patients with (*n* = 6) and without COPD (*n* = 10). No difference between the different phyla was observed (*p* > 0.05)

An overview of the clinical characteristics of the study subjects showed that all individuals were between 40 and 75 years of age with a majority on highly active antiretroviral therapy (HAART) at the time of assessment [Table 1]. The total 16S concentration in each subject following background negative control subtraction was 0.42 ± 1.39 16S/ng of DNA (mean ± standard deviation). The Shannon Diversity was 2.13 ± 0.54, OTU richness was 37.52 ± 11.83, and evenness was 0.59 ± 0.13 for this patient population (mean ± standard deviation). The distribution of Shannon Diversity, OTU richness, and evenness can be found in the Additional file 1: Table S1. On a cursory overview, the phyla distribution seems to be quite different than the experimental background negative controls. However, there was only a significant difference in the relative abundance of the Actinobacteria phylum, following Bonferroni correction, between the HIV group and background negative controls (*p* = 0.003) [Fig. 1a]. This would suggest that apart from the Actinobacteria phylum the other phyla distributions are similar to the background negative controls. A total of 23.8 % of HIV subjects contained OTUs that aligned to *Tropheryma*.

**Table 1:** An overview of clinical traits of HIV infected patients sampled in this study

Age	Vital Status	Current VL	Current CD4	Bronchoscopy Indication	Smoking Status	Pack-Years	Current HAART	CT Emphysema	CT Bronchiectasis	FEV1 (L)	FEV1/FVC (%)
60-69	Alive	<40	400–499	Cancer	Current	30	Yes	Yes	Yes	1.53	33.04
50–59	Alive	1000–9999	100–199	Pneumonia	Current	39	No	Yes	No	1.11	90.00
70–79	Alive	<40	500–599	Cancer	Current	130	Yes	Yes	No	2.99	70.55
50–59	Alive	<40	600–699	Cancer	Current	12.5	Yes	Yes	No	3.35	56.63
50–59	Alive	40–1000	500–599	Cancer	Current	37.5	Yes	Yes	No	2.71	64.18
70–79	Alive	10000–99999	200–299	Cancer	Current	30	No	No	No	N/A	N/A
50–59	Alive	<40	900–999	Cancer	Current	15	Yes	No	No	3.33	70.95
50–59	Alive	10000–99999	200–299	Pneumonia	Current	19.5	No	Yes	No	N/A	N/A
60–69	Alive	<40	700–799	Cancer	Past	20	Yes	Yes	No	2.54	59.86
60–69	Alive	<40	800–899	Cancer	Past	3	Yes	Yes	No	2.87	71.44
60–69	Alive	40–999	200–299	Cancer	Past	45	Yes	No	No	2.41	76.80
60–69	Alive	<40	≥1000	Bronchiectasis	Past	12	Yes	No	Yes	3.06	70.57
60–69	Alive	<40	300–399	Pneumonia	Past	75	Yes	No	Yes	2.47	85
40–49	Alive	<40	100–199	Cancer	Current	30	Yes	Yes	No	2.41	51.56
40–49	Alive	10000–99999	100–199	Pneumonia	Current	115	Yes	Yes	Yes	2	78.28
50–59	Alive	<40	400–499	Cancer	Past	90	Yes	Yes	No	3.33	75.76
70–79	Deceased	<40	400–499	Cancer	Past	20	Yes	Yes	Yes	2.75	69.09
60–69	Deceased	<40	100–199	Cancer	Past	4	Yes	No	Yes	2.45	72.86
60–69	Deceased	<40	100–199	Cancer	None	0	Yes	Yes	No	N/A	N/A
50–59	Deceased	<40	100–199	Pneumonia	Current	N/A	Yes	Yes	No	N/A	N/A
40–49	Deceased	≥100000	<100	Cancer	None	0	No	Yes	No	N/A	N/A

### Airway microbiome comparisons between those with and without COPD by spirometry

**Fig. 2:**
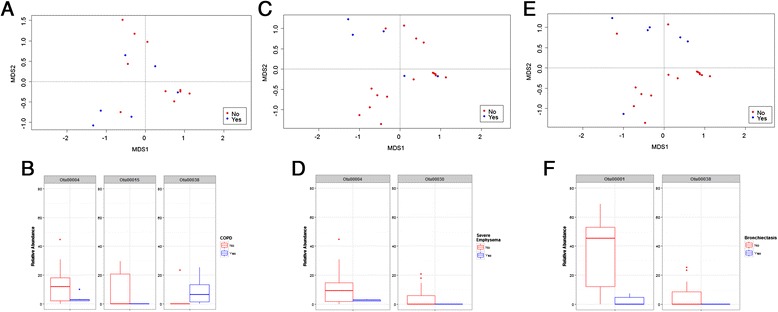
Bacterial community composition and COPD status, severe emphysema, and bronchiectasis. **a** Non-metric multidimensional scaling analysis of individuals with and without COPD, PERMANOVA = 0.10. **b** Boxplot of the relative abundance of each of the discriminative OTUs for COPD status. **c** Non-metric multidimensional scaling analysis of individuals with and without severe emphysema, PERMANOVA = 0.06. **d** Boxplot of the relative abundance of each of the discriminative OTUs for severe emphysema. **e** Non-metric multidimensional scaling analysis of individuals with and without bronchiectasis, PERMANOVA = 0.04. **f** Boxplot of the relative abundance of each of the discriminative OTUs for bronchiectasis

There was no difference in Shannon Diversity, evenness, and OTU richness between HIV patients with and without COPD (*p* > 0.05). The diagnosis of COPD had no influence on the phyla observed (*p* > 0.05) [Fig. 1b]. However, in COPD, there was a trend towards greater abundance of the Actinobacteria and Proteobacteria phyla. Using a Bray-Curtis dissimilarity matrix and Non-Metric Multidimensional Scaling (NMDS) with PERMANOVA, we found no significant difference in the bacterial community composition between those with and without COPD (PERMANOVA = 0.10) [Fig. 2a]. However, analysis of specific OTUs in relation to COPD status revealed 3 OTUs that were able to discriminate HIV patients with and without COPD [Fig. 2b]: OTU4 (Pasteurellaceae), OTU15 (Brachybacterium), and OTU38 (Yersinia). In COPD samples, there was a paucity of OTU4 and OTU15, and a slight enrichment of OTU38 [Fig. 2b]. Ribosomal database classifier [24, 25] revealed that OTU4 contained sequences of bacteria in the genus for *Haemophilus*

### Airway microbiome comparisons based on CT presence of emphysema or bronchiectasis

We did not detect a significant difference in the bacterial community composition based on severe emphysema status that was detected on CT scans (PERMANOVA = 0.06) [Fig. 2c]. However, there were two OTUs that discriminated samples from those with and without emphysema: OTU4 (*Pasteurellaceae-Haemophilus*) and OTU30 (*Pedobacter*). There was no difference in bacterial community composition in relation to emphysema distribution (whether centrilobular or paraseptal) or across respiratory bronchiolitis severity (both PERMANOVA > 0.10) (data not shown).

In those with bronchiectasis on CT, the bacterial community composition was also not significantly different from those without any bronchiectasis (PERMANOVA = 0.04) [Fig. 2e]. Two OTUs were important for this discrimination: OTU1 (*Prevotella*), and OTU38 (*Yersinia*).

### The impact of acute lung infection on airway microbiome and host responses

We found no significant difference in the bacterial community composition between those with and without pneumonia (PERMANOVA = 0.30) probably because the bronchoscopic samples were obtained from the lung contralateral to the site of active infection. We also found no significant differences in bacterial community composition between those with CD4 counts above or below 200 (PERMANOVA = 0.84), across smoking status (current, past, or never smokers; PERMANOVA = 0.37), or whether Tropheryma was detected or not (PERMANOVA = 0.16) [Additional file 1: Figure S1–S5].

### Significant pathways in WGCNA modules that correlated with the bacterial microbiome

The power measurement of 6 was used to create the gene co-expression network and a single sample was excluded since it was an extreme outlier [21, 22] [Additional file 1: Figures S6 and S7]. This sample was considered an outlier since on the hierarchical clustering of the gene expression data it formed its own unique branch on the tree versus all other samples [Additional file 1: Figure S7]. DAVID was used to assess the most relevant pathways involved in the WGCNA modules that correlated with the bacterial microbiome. In total there were 14/23 (60.8 %) gene expression modules that correlated with at least one measure of the bacterial microbiome [Table 2]. Most interesting were the immune pathways identified by the Tan, Red, Pink, and Green Yellow modules and the cilia pathways represented by the Green module [Table 2].

**Table 2:** An overview of significant gene expression module pathways

Gene Expression Module	Number of Genes	Pathway Identified	FDR
Tan	201	Lysosome, Immune Response, Plasma Membrane	<5.0 × 10-4
Red	554	Immune Response, Defense Response, Inflammatory Response, Response to Wounding	<1.0 × 10-12
Midnight Blue	82	Magnesium Ion Binding	<0.05
Green	791	Cilia	<2 × 10-4
Turquoise	6050	Intracellular Organelle, Membrane-Enclosed Lumen	<2 × 10-7
Dark Green	43	None Identified	N/A
Black	452	Cell to Cell Signaling, Cell Membrane	<1.0 × 10-5
Magenta	365	Oxidation/Reduction, Microsomes	<2.0 × 10-2
Pink	427	Immune Response, Immunoglobulion, Antigen Presentation	<1.0 × 10-3
Brown	1274	Glycoprotein, Plasma Membrane, Immune Response	<1.0 × 10-6
Blue	5675	Nucleus, Transcription Regulation, Nuclear Lumen	<2 × 10-4
Grey	5861	Olfactory Transduction	<4.9 × 10-42
Green Yellow	245	Immunoglobulin, Antigen Processing and Presentation	<1 × 10-4
Light Green	63	None Identified	N/A

### WGCNA of alpha diversity and phyla with gene expression

**Fig. 3:**
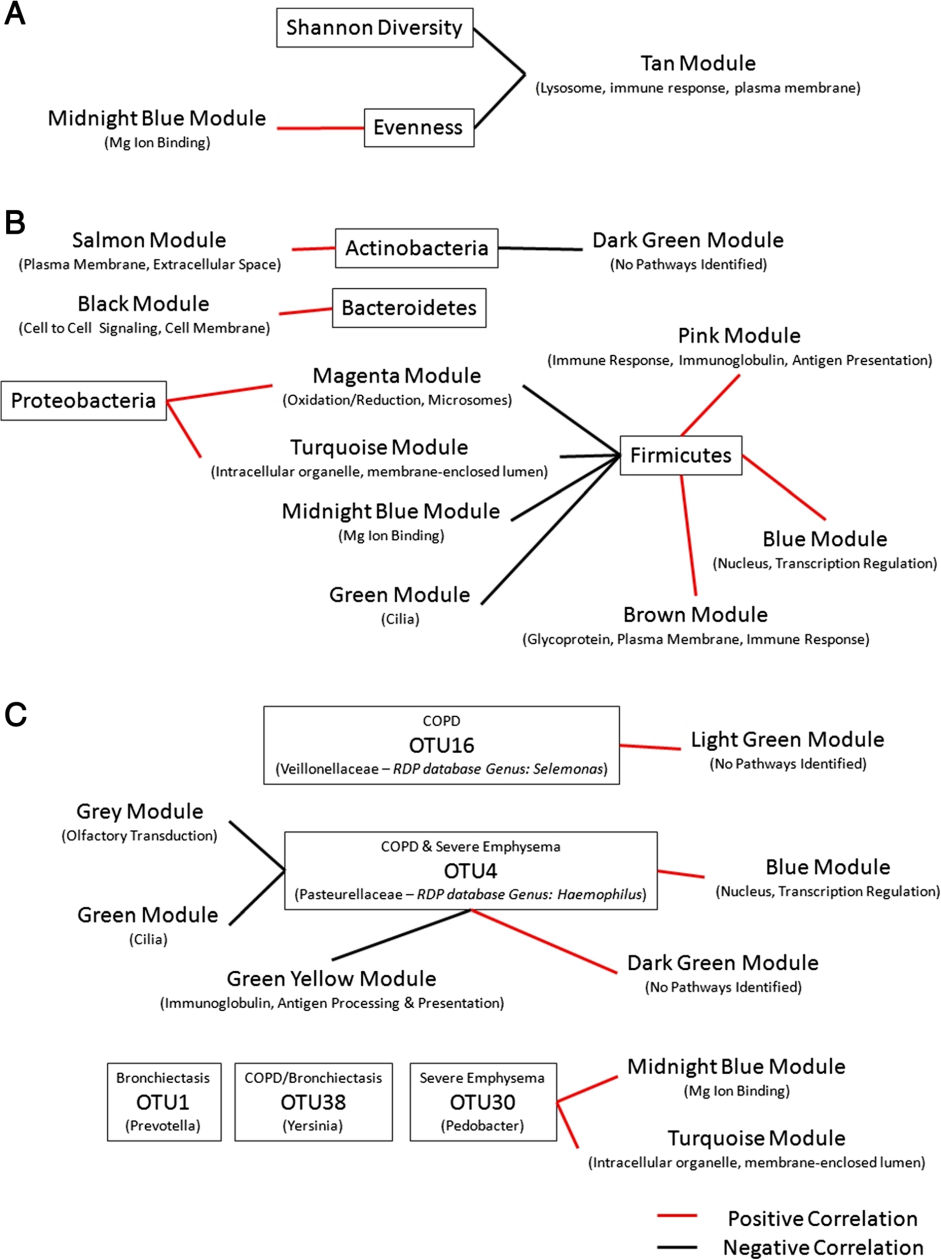
Network of the module correlations with bacterial microbiome measures. **a** Gene expression modules and alpha diversity measures. **b** Gene expression modules and bacterial phyla. **c** Gene expression modules and important OTUs for COPD, severe emphysema, and bronchiectasis. In brackets under each module is a brief description of pathways identified by DAVID for genes in the module. Red represents significant positive correlations while black represents significant negative correlations

Our analysis revealed a number of gene expression modules that correlated with the Firmicutes phylum. It was the only group that had significant correlations with any modules after FDR correction (two negative and two positive correlations). The negatively correlated modules were Green (FDR = 0.037, *p* = 4 × 10-4), Midnight Blue (FDR = 0.037, *p* = 8 × 10-4). The positively correlated modules were, Brown (FDR = 0.037, *p* = 8 × 10-4), and Blue (FDR = 0.037, *p* = 5 × 10-4) [Fig. 3 and Additional file 1: Figure S9].

When looking at those that had a *p* value under 0.05 but not an FDR under 0.05 there were additional correlations that have been summarized in Fig. 3. Briefly, the Tan module may be negatively correlated with both Shannon Diversity and evenness (FDR = 0.283, *p* = 0.02 and FDR = 0.368, *p* = 0.03 respectively) [Fig. 3a]. Evenness may also be correlated with the Midnight Blue module (FDR = 0.184, *p* = 0.009) [Fig. 3a]. The Proteobacteria phylum may also be positively correlated with the Magenta module (FDR = 0.283, *p* = 0.02) and the Turquoise module (FDR = 0.368, *p* = 0.03) [Fig. 3b].

### WGCNA of the important OTUs and gene expression

No modules and OTUs found to be predictive by random forest for COPD, severe emphysema, or bronchiectasis were found above an FDR of 0.05. are described in the Additional file 1: Figure S10. However, some correlations between modules and these OTUs occurred with a p-value under 0.05. This included OTU4 [Fig. 3c] which was negatively correlated with the Grey (FDR = 0.941, *p* = 0.04), Green (FDR = 0.941, *p* = 0.04), and Green Yellow (FDR = *p* = 0.03) modules. It was also positively correlated with the Blue (*p* = 0.004) and Dark Green (*p* = 0.04) modules.

## Discussion

The interplay between the microbiome and host gene expression is increasingly recognized as a key element of health and disease. Our study extensively examined the relationship between the bacterial microbiome and host gene expression from bronchial epithelial cells taken from the same small airways of patients infected with HIV. We found that the small airway microbiome of HIV-infected patients demonstrated only modest differences in the global bacterial community composition compared with background negative controls [Fig. 1]. However, we did not find any significant differences in the global airway bacterial composition between those with and without COPD, between those with elevated or reduced CD4 counts, between those with bronchiectasis, or between those with and without emphysema on CT scans [Fig. 2]. However, when we investigated individual OTUs using an unocrrected PERMANOVA threshold of 0.10 or below, we discovered OTU signatures that were distinct for those with COPD (measured by spirometry), severe emphysema (detected on CT), and bronchiectasis. Spirometry-based COPD was associated with OTU4, OTU15, and OTU38, severe emphysema was associated with OTU4 and OTU30, while bronchiectasis was associated with OTU1 and OTU38. More importantly, we found that measures of the airway microbiome including alpha diversity measures, phyla, and OTUs, were significantly related to distinct host response in the same airway as captured by gene expression modules. Many of these modules involved immune and inflammatory responses, cell signaling, and cilia pathways suggesting immunomodulatory role of the airway microbiota in the host’s ability to process and remove irritants and aeropathogens. Additional work will be needed to validate this hypothesis.

Our findings may be consistent with previous studies on the lung microbiome in HIV, which found *Tropheryma whipplei* as a discriminative bacterium in bronchoalveolar lavage fluid (BALF), occurring in 13.4 % of HIV subjects versus only 1.3 % of HIV-uninfected subjects [4]. In our study, which used bronchial brushes rather than BALF, we demonstrated the presence of *Tropheryma* in 23.4 % of the samples. Although these data are in line with the previous literature, additional molecular studies such as a qPCR assay targeting a gene specific for the species would be needed to confirm that the *Tropheryma* we identified was indeed *T.whipplei*. We extend the previous findings by characterizing the host gene expression response to the bacterial microbiome. For instance, we found that Shannon Diversity and evenness were negatively correlated with genes involved with lysosome formation and immune response. This finding is consistent with the evolving concept that reduction in bacterial diversity is associated with an elevated risk of clinical infection and increased inflammatory response by the host [26, 27]. It should be noted that certain organisms independent of their numbers, are more likely to elicit an inflammatory response compared with others that are less “pathogenic”. For instance, although in our study we found that bacteria in the Actinobacteria phylum were significantly more abundant in HIV lungs than in the background negative environmental controls, these bacteria were not significantly associated with gene expression modules. In contrast, bacteria in the Firmicutes phyla (though less abundant compared with Actinobacteria) were significantly associated with several different gene expression modules. Firmicutes were negatively related to pathways governing cilium and positively associated with gene expression modules associated with immune response and transcription regulation. When we explored all correlations that had a *p*-value under 0.05 [Fig. 3] the Proteobacteria phylum was positively associated with gene expression pathways related to oxidation/reduction and intracellular orgnaelles, whereas the Firmicutes phylum was negatively associated with these pathways. This data is consistent with a previous study which reported a natural antagonism between the Firmicutes and Proteobacteria phyla in the oropharynx [28]. These data are also consistent with the evolving concept that the lung microbiome is propagated by upper airway seeding [29, 30]. We speculate that the host immune response is regulated in the HIV lung by the seeding of certain organisms from the upper airways into the lower airway tract. We posit that the predominance of Firmicutes phylum leads to a heightened inflammatory state. Additional studies into the host interactions with the bacterial microbiome within the lung will need to be completed to confirm this hypothesis.

Most intriguingly, we found that OTU4 (*Pasteurellaceae-Haemophilus*) was predictive of both COPD (by spirometry) and severe emphysema (by CT). Previous studies suggest that *Haemophilus influenzae* is an important pathogen in COPD [31] and a recent study using whole lung tissue has shown that this organism is found in control subjects but not in patients with GOLD 4 severity [7]. Consistent with this observation, in our study *Haemophilus spp* was found in airways of patients without COPD by spirometry and without significant emphysema on CT scan [Figs. 2 and 3], although there were no correlations under and FDR < 0.05 when exploring OTU correlations with gene expression modules. Intriguingly, those correlations that were under a p value of 0.05 showed that OTU4 negatively correlated with both pathways involved with cilia and antigen processing and presentation [Fig. 3]. This raises the tantalizing hypothesis that up-regulation of immune genes which activate the adaptive immune processes may enable processing and removal of *Haemophilus spp* in the airways. However, this result would need to be validated in a study with more power to asses this relationship. Up-regulation of genes involved in cilia may have a similar effect. We speculate that COPD airways have altered immune and/or cilia function that may prevent effective clearance of *Haemophilus spp*. Additional work will be needed to validate this hypothesis.

There are several limitations to this study. First, the findings pertain exclusively to HIV-infected patients. Thus it is possible that these OTUs may not help to distinguish COPD, severe emphysema, or bronchiectasis in HIV-uninfected patients. However, a recent study suggests that the bacterial microbiome between HIV-uninfected patients and HIV patients on successful antiretroviral therapy may be similar [5]. Second, no oral wash was performed prior to the bronchoscopy. This could have led to minor contaminations of the bronchial brush samples. However, it was reassuring that the findings of the present study were consistent with others that used mouth rinsing procedures [6, 29, 32]. Thirdly, all patients enrolled in this study had a clinical indication for bronchoscopy. While great care was taken to sample epithelial cells from uninfected regions of the lung, and far away from nodules or masses, our results could be confounded by these underlying conditions. However, we did not find the bacterial species identified by routine clinical culture in those patients with a diagnosis of pneumonia in the analysis of 16S, which would support that sampling was indeed from the unaffected portions of the lung. This would explain the fact that there was no difference in the bacterial community composition between those with and without pneumonia. Another limitation is that we were unable to validate the microarray expression results with RT-PCR due to the large size of many of the modules. Thus it is possible that some of the genes within the modules are not accurate. However, a module is based on more than one gene and in order for a module to be wrong the majority of gene expression values within it would have to be incorrect. Finally, it is possible that some of the gene expression could be accounted for by infiltrating immune cells that were taken along with the epithelial cells during sampling. We cannot conclusively rule this possibility out but samples in this study were obtained away from locations with signs of clear inflammation. Future studies in which the bronchial epithelial cell microbiome is assessed in asymptomatic HIV-infected individuals would help to clarify the relationships between the microbiome and host response, and in certain pulmonary phenotypes.

Overall, this study provides a preliminary investigation into the host gene expression interaction with the bacterial microbiome in the small airways of HIV infected individuals. It supports the hypothesis that diversity and evenness of the community are important in modulating inflammatory responses of the host. This study also shows how bacteria in some phyla and OTUs may be important in disease pathogenesis by modifying either the host response and/or ecological niche areas. Our work supports the possibility that specific interactions between the bacterial microbiome and host cells within the airways of the lung occur and may be associated with distinct disease phenotypes; these findings would require additional studies for validation.

## Conclusions

In summary this study demonstrates that the bacterial microbiome and host gene expression may interact with one another in individuals with HIV infection. It identifies pathways, such as the mucocillary transport system, as important in the interaction between host and bacterial microbiome. However, more research into this specific area needs to be accomplished to confirm these results and observations.

## Additional file

Additional file 1: Table S1. Alpha diversity measures used in the analysis. **Figure S1.** NMDS of HIV patients based on pneumonia status. **Figure S2.** NMDS of HIV patients based on CD4 counts below 200. **Figure S3.** NMDS of HIV patients based on smoking status. **Figure S4.** NMDS of HIV patients based on whether Emphysema was present on CT scan. **Figure S5.** NMDS of HIV patients based on whether Tropheryma was present. **Figure S6.** Power threshold calculation for WGCNA. **Figure S7.** Outlier detection for WGCNA. A single sample was removed (BIDC25) since it clustered quite differently than the rest of the data set. **Figure S8.** Heatmap showing that BIDC25 is also very different than other samples versus traits of interest. **Figure S9.** Module trait relationship with alpha diversity measures and phyla. Green squares represent negative correlations while red squares represent positive correlations. The tow numbers displayed in each square is the R-value correlation and P-value (number in brackets) respectively. **Figure S10.** Module trait relationship with the important OTUs identified. Green squares represent negative correlations while red squares represent positive correlations. The two numbers displayed in each square is the R-value correlations and P-value (number in brackets respectively). (DOC 430 kb)

## Abbreviations

16S: 16 Svedberg
BC: British Columbia
BIDC: Internal naming system for patient samples
COPD: Chronic Obstructive Pulmonary Disease
CT: Computed Tomography
DAVID: Database for Annotation Visualization and Integrated Discovery
ddPCR: Droplet digital Polymerase Chain Reaction
FDR: False discovery rate
FEV1: Forced expiratory volume in 1 second
FVC: Forced vital capacity
HAART: Highly active antiretroviral therapy
HIV: Human immunodeficiency virus
NCBI: Nationa Center for Biotechnology Information
Neg: Negative
NMDS: Non-metric multidimensional scaling
OTU: Operational taxonomic unit
PCR: Polymerase chain reaction
PERMANOVA: Permutation analysis of variance
RMA: Robust multi-array average
rRNA: Ribosomal ribonucleic acid
SPH: Saint Paul’s Hospital
SRP: Sequence read project
UBC: University of British Columbia
WGCNA: Weighted gene co-expression network analysis
