# Supplementary material for: The bronchial epithelial cell bacterial microbiome and host response in patients infected with human immunodeficiency virus
Source: BMC Pulm Med. 2016 Nov 9;16:142. doi: 10.1186/s12890-016-0303-4 (PMC5103452; doi:10.1186/s12890-016-0303-4)
Supplement: Additional file 1: Table S1. — Alpha diversity measures used in the analysis. Figure S1. NMDS of HIV patients based on pneumonia status. Figure S2. NMDS of HIV patients based on CD4 counts below 200. Figure S3. NMDS of HIV patients based on smoking status. Figure S4. NMDS of HIV patients based on whether Emphysema was present on CT scan. Figure S5. NMDS of HIV patients based on whether Tropheryma was present. Figure S6. Power threshold calculation for WGCNA. Figure S7. Outlier detection for WGCNA. A single sample was removed (BIDC25) since it clustered quite differently than the rest of the data set. Figure S8. Heatmap showing that BIDC25 is also very different than other samples versus traits of interest. Figure S9. Module trait relationship with alpha diversity measures and phyla. Green squares represent negative correlations while red squares represent positive correlations. The tow numbers displayed in each square is the R-value correlation and P-value (number in brackets) respectively. Figure S10. Module trait relationship with the important OTUs identified. Green squares represent negative correlations while red squares represent positive correlations. The two numbers displayed in each square is the R-value correlations and P-value (number in brackets respectively). (DOC 430 kb) [file 12890_2016_303_MOESM1_ESM.doc]

**The Host Response to the Bronchial Epithelial Cell Bacterial Microbiome in Human Immunodeficiency Virus Infection**

Marc A. Sze, Stella Xu, Janice M. Leung, Tawimas Shaipanich, Aida Moghadam, Marianne Harris, Silvia Guillemi, Sunita Sinha, Corey Nislow, Darra Murphy, Cameron Hague, Jonathon Leipsic, Julio Montaner, Don D. Sin, S.F. Paul Man

**Additional file 1**

**Emphysema Severity Scoring:**

A score based on the extent of emphysema as a percentage of total lung volume assigned to each of the five lobes plus the lingula: 0=0% emphysema, 1 =1-25% emphysema, 2=26-50% emphysema, 3=51-75% emphysema, 4=76-100% emphysema. A total score was calculated from the summation of the five lobes and lingua scores, with a score ≥ 4 considered severe emphysema.

**Bronchoscopy and specimen collection:**

After induction of conscious sedation, a flexible bronchoscope was passed with minimal suction through the subject's oropharynx. Topical 1% lidocaine was applied throughout the vocal cords, trachea, and main bronchi. Cytologic brushings were obtained in the upper lobe small airways while avoiding areas of pathology apparent on CT imaging (such as consolidations, lung nodules or masses, and areas of bronchiectasis). Two brushes were taken from adjacent subsegmental airways, with one brush preserved in Cytolyt (Cytyc, Marlborough, MA) for DNA preservation and the other brush preserved in Trizol (Qiagen, Venlo, the Netherlands) for RNA preservation. Brushes contaminated with blood were discarded. DNA and RNA were also extracted from background negative environmental control samples (water samples that were poured over sterile, unused cytologic brushes and collected in the bronchoscopy suite).

**Touchdown PCR Cycle Conditions:**

95ºC for 2 minutes

(95ºC for 20 seconds, 60ºC/54ºC for 15 seconds*, 72ºC for 1 minute 30 seconds) x 20

* Temperature decrease of 0.3ºC at annealing temperature each cycle to 54ºC at cycle 20

(95ºC for 20 seconds, 55ºC for 15 seconds, 72ºC for 1 minute 30 seconds) x 20

72ºC for 5 minutes

**Weighted Gene Co-Expression Network Analysis**

Papers on the theory of Weighted Gene Co-Expression Network Analysis (WGCNA) can be found at the following links <http://labs.genetics.ucla.edu/horvath/CoexpressionNetwork/> and an in depth tutorial on how to perform a WGCNA can be found at labs.genetics.ucla.edu/horvath/CoexpressionNetwork/Rpackages/WGCNA/Tutorials/. Briefly, the WGCNA is often used to explore the overall functionality of genes on the system level. The idea is to imagine genes as nodes and look for node connections if subsequent genes are correlated within the same sample. The weighted component comes from not strictly defining a gene co-expression as completely “yes” or “no”. A cutoff for co-expressed genes in this data set was defined by using a scale-free topology criterion.

Table S1: Alpha diversity measures used in the analysis

| H | S | J | FEV1/FVC (%) | Sample ID |
| --- | --- | --- | --- | --- |
| 1.827656386 | 28 | 0.548482658 | 33.04 | BIDC2 |
| 2.383259023 | 34 | 0.675841 | 90.00 | BIDC4 |
| 1.677926776 | 24 | 0.527973051 | 70.55 | BIDC7 |
| 2.458338269 | 34 | 0.697131859 | 56.63 | BIDC8 |
| 2.024068311 | 25 | 0.628812176 | 64.18 | BIDC9 |
| 2.40341473 | 30 | 0.706637828 | N/A | BIDC10 |
| 2.446779974 | 41 | 0.658875048 | 70.95 | BIDC14 |
| 1.868381067 | 46 | 0.488001062 | N/A | BIDC15 |
| 2.522035194 | 38 | 0.693326586 | 59.86 | BIDC16 |
| 2.383808649 | 41 | 0.641917972 | 71.44 | BIDC20 |
| 2.579635616 | 60 | 0.630048492 | 76.80 | BIDC21 |
| 1.802559063 | 32 | 0.520108604 | 70.57 | BIDC22 |
| 1.448226561 | 27 | 0.439410875 | 85 | BIDC23 |
| 1.918321431 | 45 | 0.503937882 | 51.56 | BIDC24 |
| 0.644411875 | 25 | 0.200197805 | 78.28 | BIDC25 |
| 3.073277892 | 56 | 0.763480592 | 75.76 | BIDC26 |
| 1.872591654 | 21 | 0.615069093 | 69.09 | BIDC1 |
| 2.956149141 | 61 | 0.719104803 | 72.86 | BIDC3 |
| 1.834402947 | 49 | 0.471348317 | N/A | BIDC17 |
| 2.338848598 | 32 | 0.674849055 | N/A | BIDC18 |
| 2.294171968 | 39 | 0.626213556 | N/A | BIDC19 |


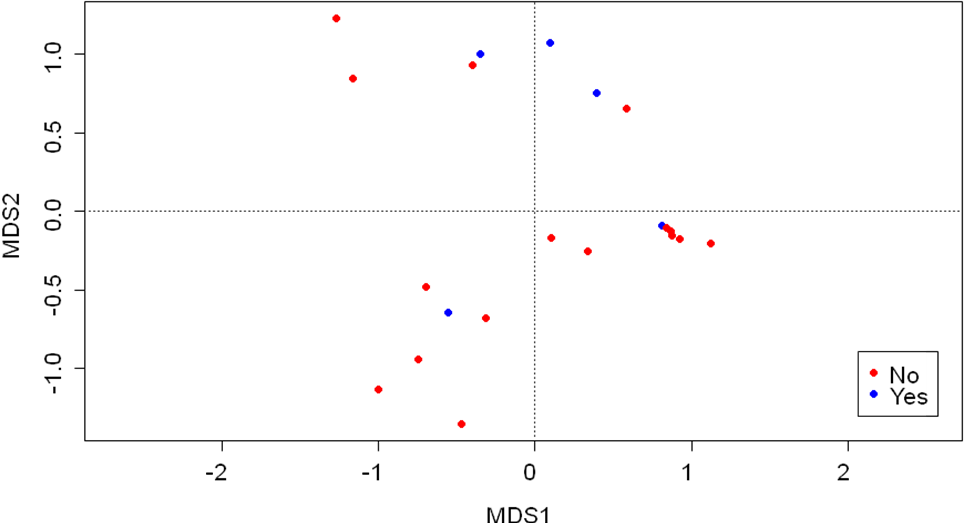


Figure S1: NMDS of HIV patients based on pneumonia status (PERMANOVA = 0.30)


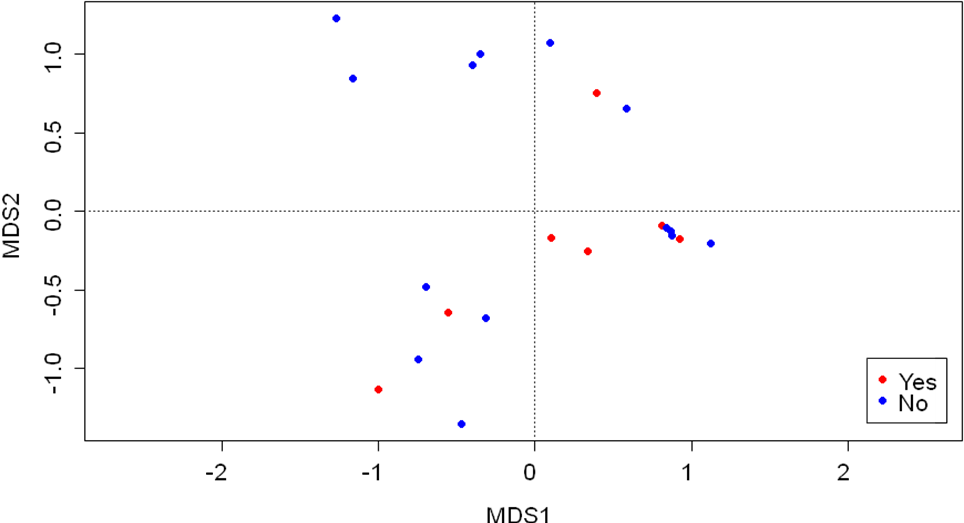


Figure S2: NMDS of HIV patients based on CD4 counts below 200 (PERMANOVA = 0.84).


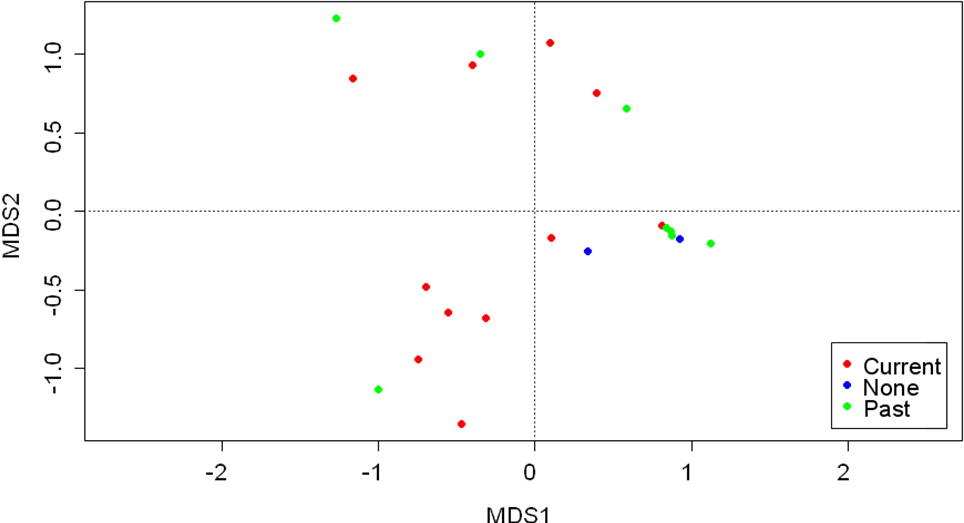


Figure S3: NMDS of HIV patients based on smoking status (PERMANOVA = 0.37).


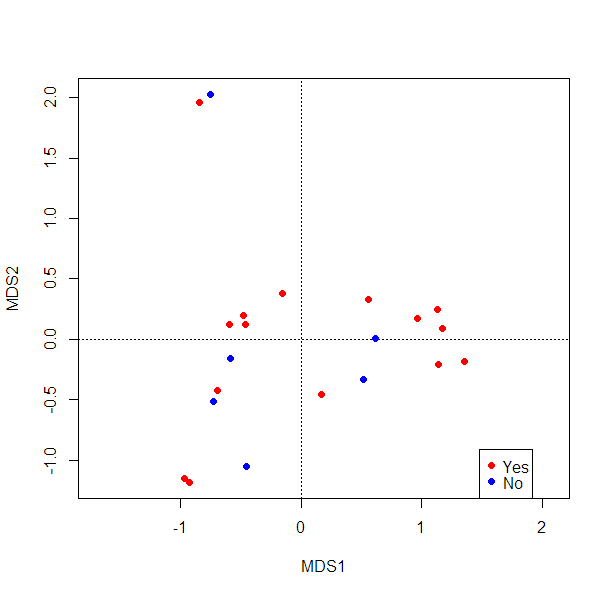


Figure S4: NMDS of HIV patients based on whether Emphysema was present on CT scan (PERMANOVA = 0.75).


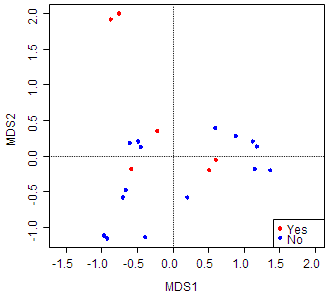


Figure S5: NMDS of HIV patients based on whether Tropheryma was present (PERMANOVA = 0.16).


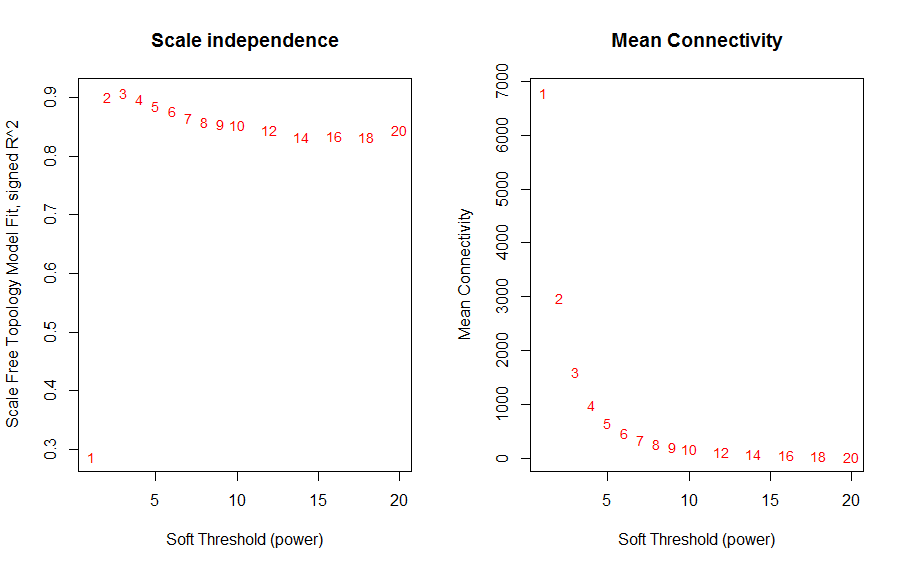


Figure S6: Power threshold calculation for WGCNA.


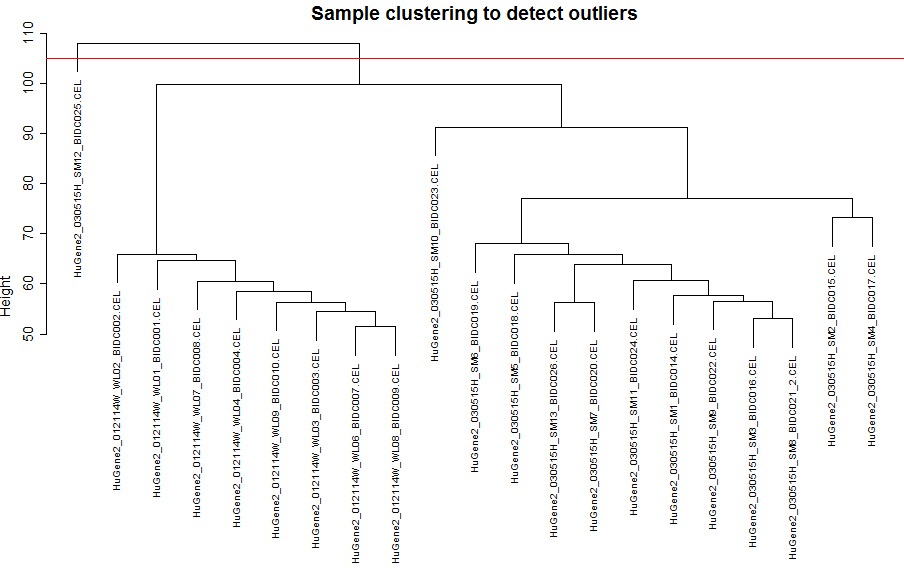


Figure S7: Outlier detection for WGCNA. A single sample was removed (BIDC25) since it clustered quite differently than the rest of the data set.


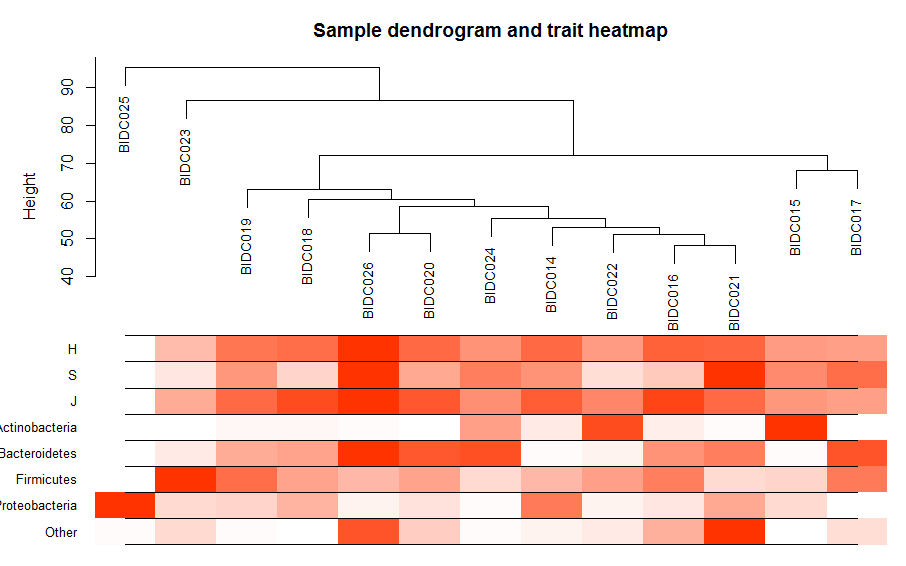


Figure S8: Heatmap showing that BIDC25 is also very different than other samples versus traits of interest.


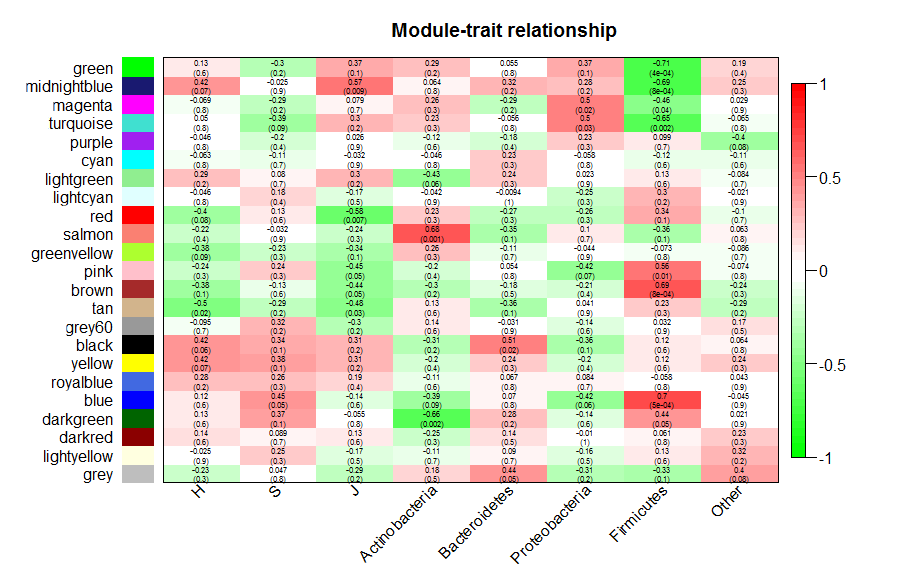


**Figure S9**: **Module trait relationship with alpha diversity measures and phyla**. Green squares represent negative correlations while red squares represent positive correlations. The two numbers displayed in each square is the R-value correlation and P-value (number in brackets) respectively.


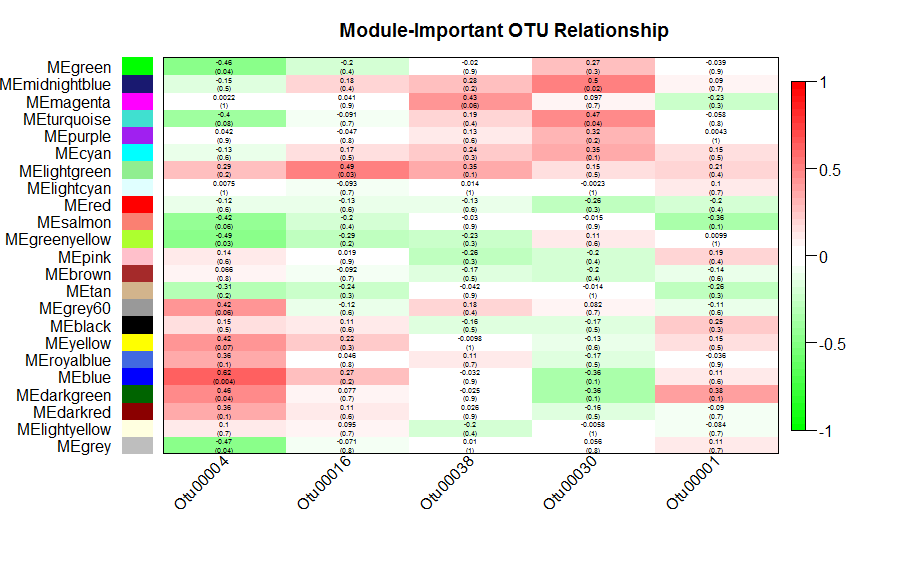


Figure S10: Module trait relationship with the important OTUs identified. Green squares represent negative correlations while red squares represent positive correlations. The two numbers displayed in each square is the R-value correlation and P-value (number in brackets) respectively.
